# Caryolene-forming carbocation rearrangements

**DOI:** 10.3762/bjoc.9.37

**Published:** 2013-02-13

**Authors:** Quynh Nhu N Nguyen, Dean J Tantillo

**Affiliations:** 1 Department of Chemistry, University of California–Davis, 1 Shields Avenue, Davis, CA 95616, USA

**Keywords:** carbocation, cycloaddition, density functional theory, mechanism, reactive intermediates, terpene

## Abstract

Density functional theory calculations on mechanisms of the formation of caryolene, a putative biosynthetic precursor to caryol-1(11)-en-10-ol, reveal two mechanisms for caryolene formation: one involves a base-catalyzed deprotonation/reprotonation sequence and tertiary carbocation minimum, whereas the other (with a higher energy barrier) involves intramolecular proton transfer and the generation of a secondary carbocation minimum and a hydrogen-bridged minimum. Both mechanisms are predicted to involve concerted suprafacial/suprafacial [2 + 2] cycloadditions, whose asynchronicity allows them to avoid the constraints of orbital symmetry.

## Introduction

**Figure 1:**
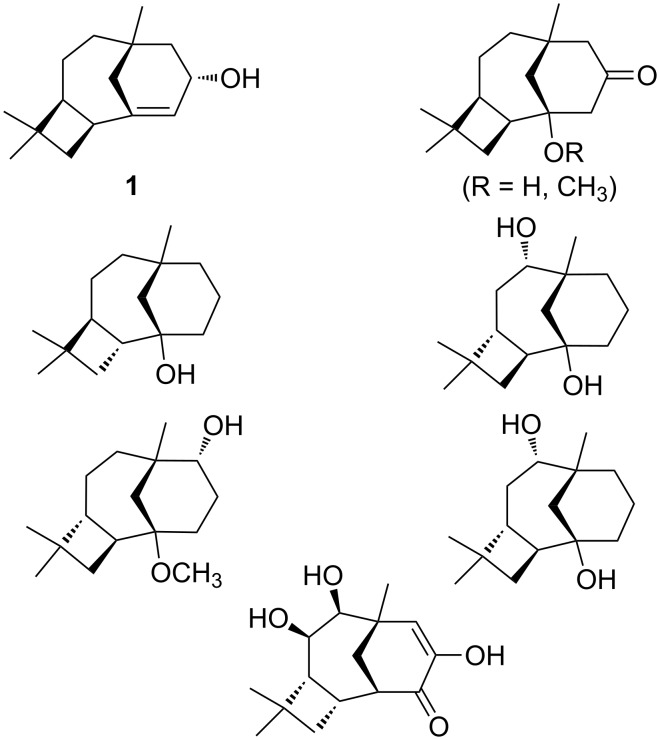
Caryol-1(11)-en-10-ol (**1**) and similar sesquiterpenoids. Note that a different atom numbering was used in the paper describing the isolation of **1** [2].

**Scheme 1:**
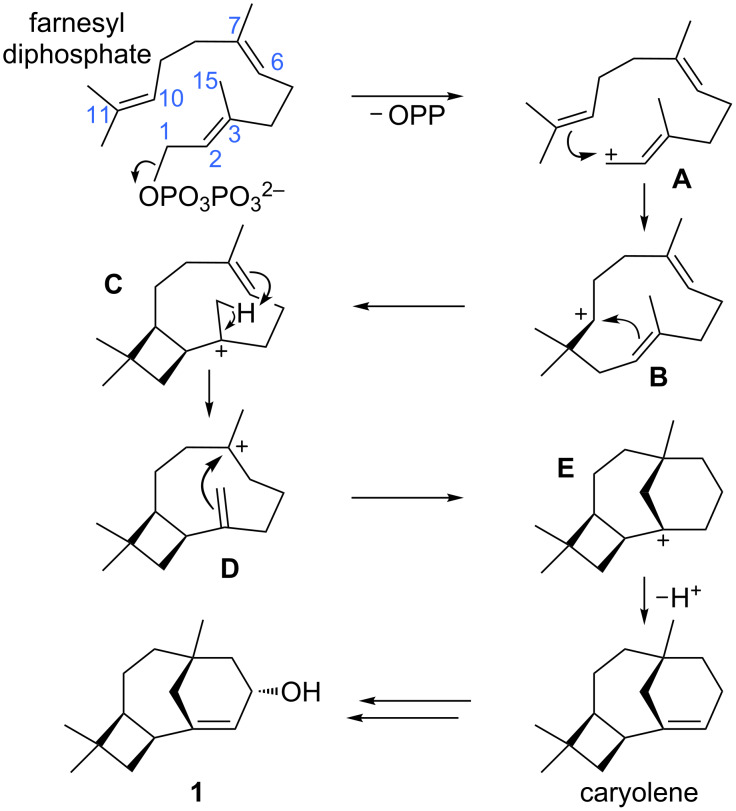
Initially proposed mechanism for caryolene (caryol-1(11)-en-10-ol, **1**) formation. Atom numbers for farnesyl diphosphate are shown. These are used in the mechanistic discussions herein; note that this numbering system differs from that generally used for caryolene and caryolenol.

The cytotoxic sesquiterpenol caryol-1(11)-en-10-ol (**1**, Figure 1) was isolated by Barrow et al. in 1988 during an investigation of antiviral/antitumor compounds from New Zealand marine invertebrates [2]. Similar sesquiterpenoids were also found in *Campanella* fungi, *Streptomyces* bacteria, *Sinacalia tangutica* plants, and *Eurypon* sponges (Figure 1) [3–6]. The carbon skeleton of **1** is unusual, not only because it contains concatenated 4-, 6-, and 7-membered rings, but also in that it bears a bridgehead double bond. As noted in the original isolation report [2], this type of bridgehead C=C bond is rare for naturally occurring compounds, since it is expected to be associated with significant strain (although it is not technically in violation of Bredt’s Rule) [7]. Intrigued by this structure, we proposed a biosynthetic mechanism for its formation (Scheme 1) and set about putting this proposal to the test using quantum chemical calculations [8].

## Results and Discussion

**Figure 2:**
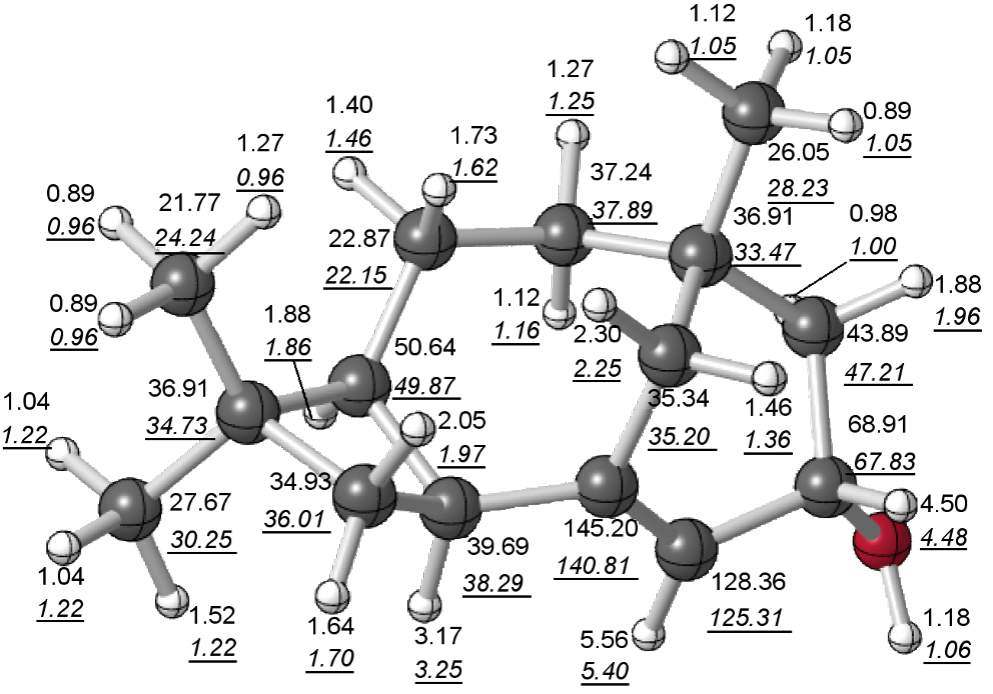
Computed (top) and experimental (bottom, underlined italics) [2] ^1^H and ^13^C chemical shifts for **1** (lowest-energy conformer found); see Experimental for details.

**Structure validation:** We first computed ^1^H and ^13^C chemical shifts for **1** to assure ourselves that the assigned structure was reasonable [9–10]. Our calculated chemical shifts and the reported data matched well (Figure 2). The mean absolute deviations between computed and experimental chemical shifts were 0.10 ppm for ^1^H and 1.96 ppm for ^13^C, and the largest deviations were 0.31 ppm and 4.40 ppm for ^1^H and ^13^C, respectively. These values are typical for structures known to be correct [11–16], giving us confidence in the original structural assignment. Interestingly, our calculations also indicate that the bridgehead C=C unit of **1** is not actually associated with much geometric strain [17].

**Proposed mechanism:** Initially, we proposed the pathway shown in Scheme 1 for caryolene formation, applying the principles derived from previous theoretical studies on terpene-forming carbocation rearrangements [8]. In this mechanism, formation of the C1–C11 bond was expected to result in secondary carbocation **B**, in analogy to previously characterized pathways to sesquiterpenes containing 11-membered rings [1,8,18–22]. The C2=C3 π-bond was then expected to attack C10 to form the 4-membered ring (see **C**), in analogy to previously proposed mechanisms for caryophyllene formation [21,23]. An intramolecular proton transfer from the C15 methyl group to the nearby C6=C7 π-bond could then generate **D**. Related intramolecular proton transfers have been described [24–33]. Attack of the resulting C3=C15 π-bond onto C7 would complete the carbon skeleton of **1**, leaving a bridgehead carbocation [34], whose deprotonation would lead to caryolene, the putative biosynthetic precursor to **1**. Despite the apparent reasonability of this proposed mechanism, our quantum chemical calculations indicated that the pathway as formulated in Scheme 1 is not energetically viable (see below).

**Figure 3:**
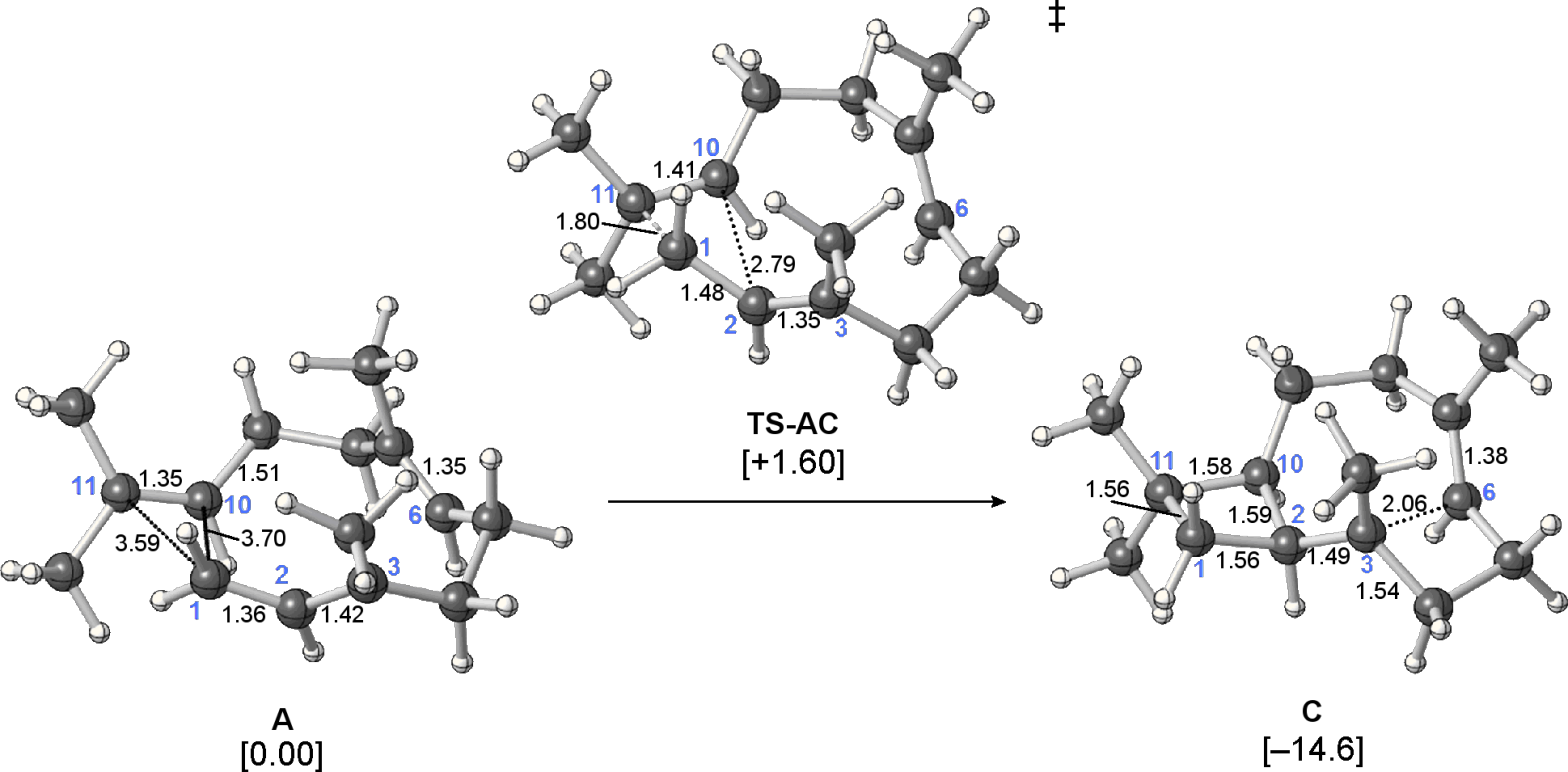
Computed minima and transition-state structure involved in the single-step conversion of **A** to **C**. Relative energies shown (kcal/mol) were calculated at the mPW1PW91/6-31+G(d,p)//B3LYP/6-31+G(d,p) level.

**Figure 4:**
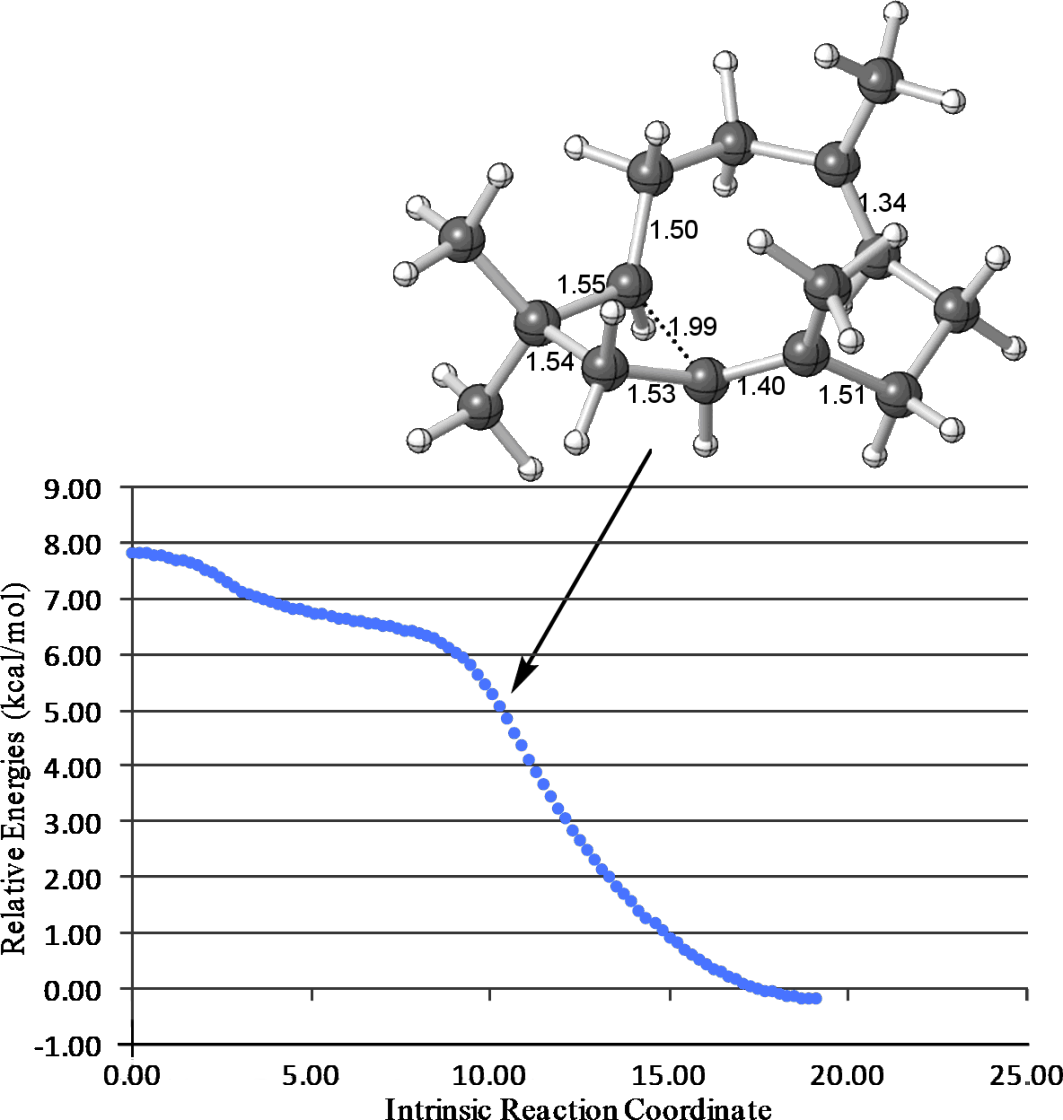
IRC from **TS-AC** toward **C**. Relative energies were calculated at the B3LYP/6-31+G(d,p) level.

**Computed mechanism:** The first deviation from the proposed mechanism in Scheme 1 was encountered in the very first step involving carbocations. We were unable to locate a minimum for **B** in a productive conformation, despite the fact that alternative conformers of this secondary carbocation had been found to be involved in pathways to pentalenene and presilphiperfolanol [18–22]. Instead, a transition-state structure connected directly (by an IRC; see Experimental for details) to farnesyl cation **A** and cyclobutylcarbinyl cation **C** was located: **TS-AC** (Figure 3 and Figure 4). This process bypasses the generation of a secondary carbocation as a minimum [22], and overall corresponds to a formally orbital-symmetry-forbidden [_π_2_S_ + _π_2_S_] cycloaddition [35–36]. Although this process is predicted to be concerted, the bond-forming events occur asynchronously [21,37–38] (note that the C1–C11 distance in **TS-AC** is approximately 1 Å shorter than the C2–C10 distance, indicating that C1–C11 bond formation leads C2–C10 bond formation) and at no point along the reaction coordinate is there significant cyclic delocalization of the sort that would be associated with a forbidden reaction. As described for other carbocation reactions [39–40], the constraints of orbital symmetry appear to have been circumvented. Interestingly, there is a shoulder (i.e., a sharp downturn) on the reaction coordinate in the vicinity of structures resembling **B** (Figure 4).

In the structure of **C** (Figure 3), C3 is quite close to C6 (only 2.06 Å away), indicating that this structure is perhaps best described as a hybrid of the tertiary cation **C** and a resonance structure with two 4-membered rings [22]. We performed a conformational search for **C** to assess whether other conformers lacking this close contact were possible, but all starting geometries examined in which the two methyl groups pointed to the same side of the ring as the first-formed cyclobutane, converged to the structure of **C** shown in Figure 3 [41].

**Scheme 2:**
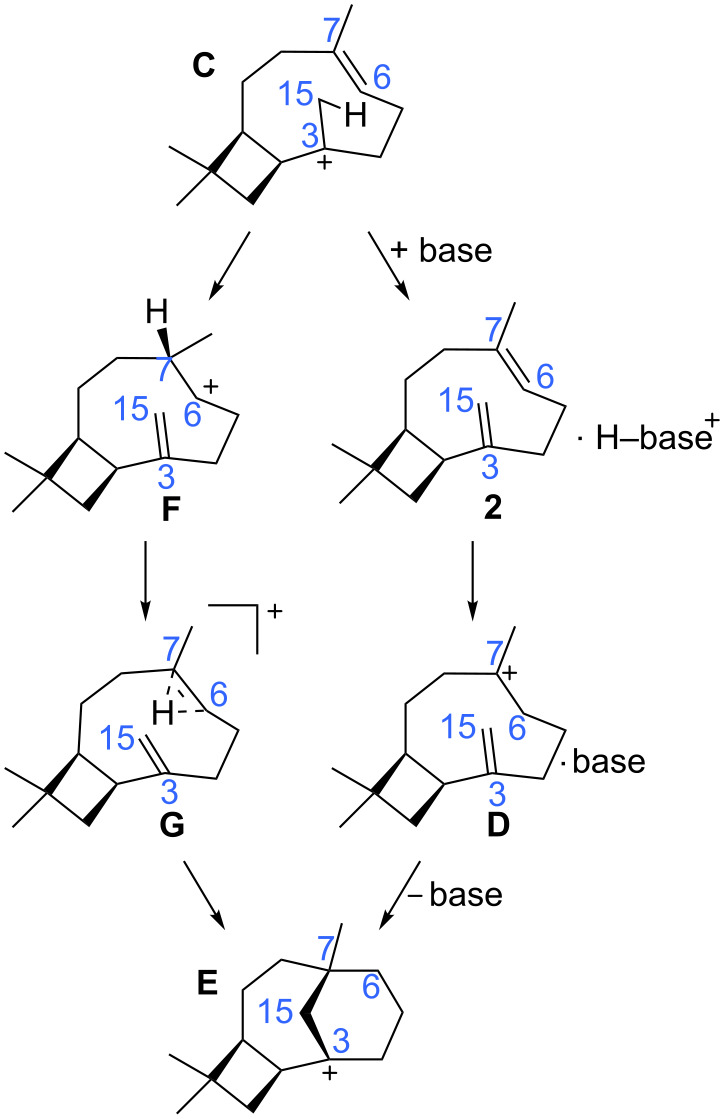
Alternative mechanisms for caryolene formation.

**Figure 5:**
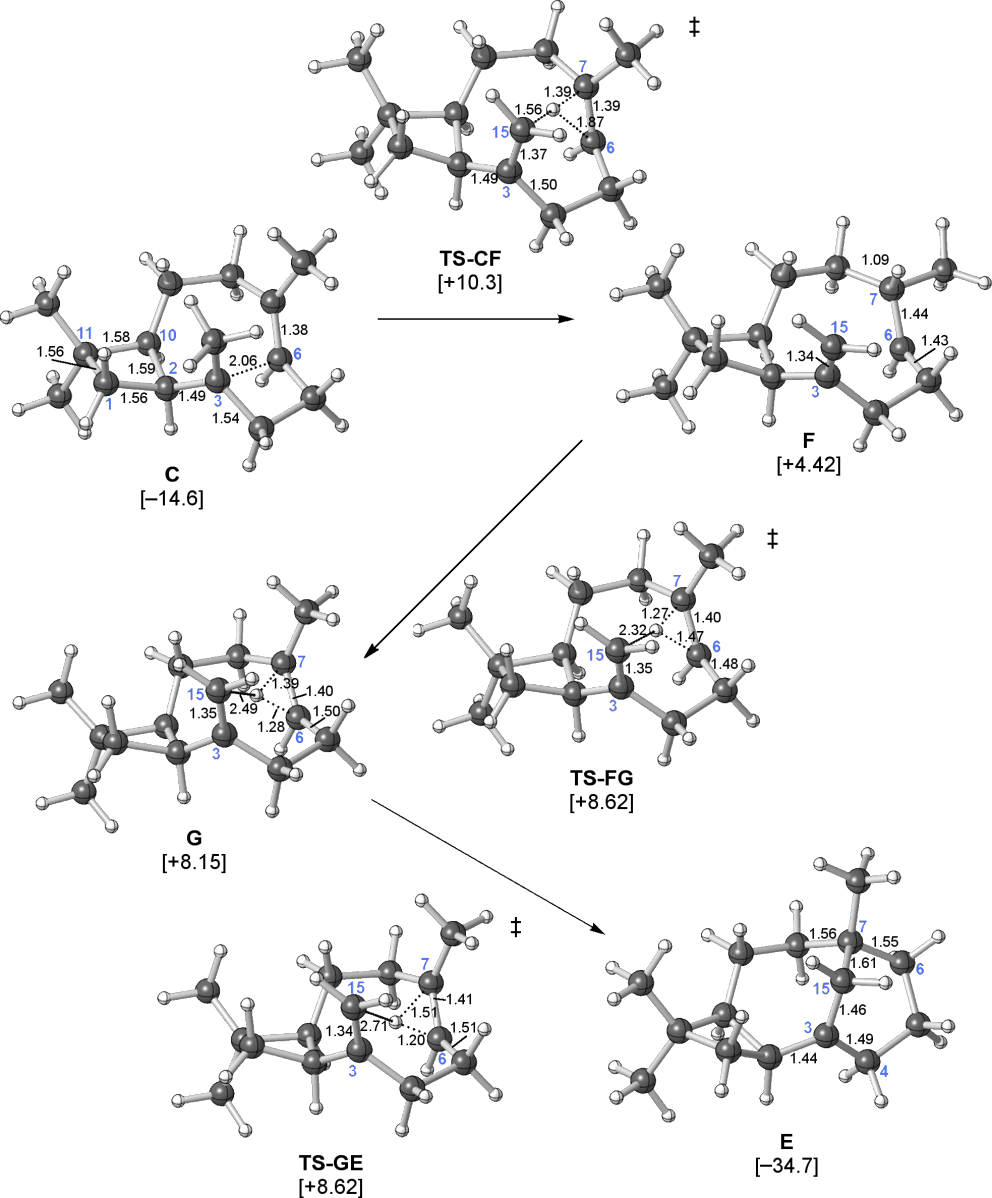
Computed pathway for the conversion of **C** to **E**. Relative energies shown (kcal/mol) were calculated at the mPW1PW91/6-31+G(d,p)//B3LYP/6-31+G(d,p) level.

Locating a pathway for the conversion of **C** to **D** (Scheme 1) also proved difficult. We expected proton transfer from the C15 methyl group to C6 of the C6=C7 π-bond to result in tertiary carbocation **D**. Surprisingly, only a transition-state structure for migration of the proton to C7 instead of C6, generating the secondary cation **F** (Scheme 2, left and Figure 5), was found. Attempts to independently locate **D** led instead to **G** (Figure 5), a nonclassical carbocation [42–46], or back to **C** [47]. Carbocation **G** contains a hydrogen bridge between C6 and C7, which also appears to interact with the nearby C3=C15 π-bond. If the interaction with the C3=C15 π-bond were stronger, this structure could be regarded as a “proton sandwich” [20,22,48]. An interesting question thus emerges about the nature of this structure: does the bridging hydrogen have hydride character (as expected for a structure resembling a transition-state structure for a 1,2-hydrogen shift) or proton character (as expected for a “proton sandwich”)? This issue was addressed through calculations of ^1^H chemical shifts, which predicted a chemical shift of +4.0 ppm for the bridging hydrogen in **G**. Although this shift is not as far downfield as that predicted for structures with bridging protons (e.g., the predicted shift for the migrating proton in **TS-CF** is +9.1 ppm and the predicted shifts for symmetric “proton sandwiches” are around +13 ppm) [20], it is well downfield of shifts predicted for hydrides involved in three-center two-electron bonding arrays (e.g., see Supporting Information File 1 for a model transition-state structure for a 1,2-hydride shift with a predicted chemical shift of +1.9 ppm) [49–51] and approximately 2 ppm downfield of its value when merely hyperconjugated in **C** (+2.6 ppm; computed partial charges paint a similar, but less clear-cut picture; see Supporting Information File 1 for details).

**Figure 6:**
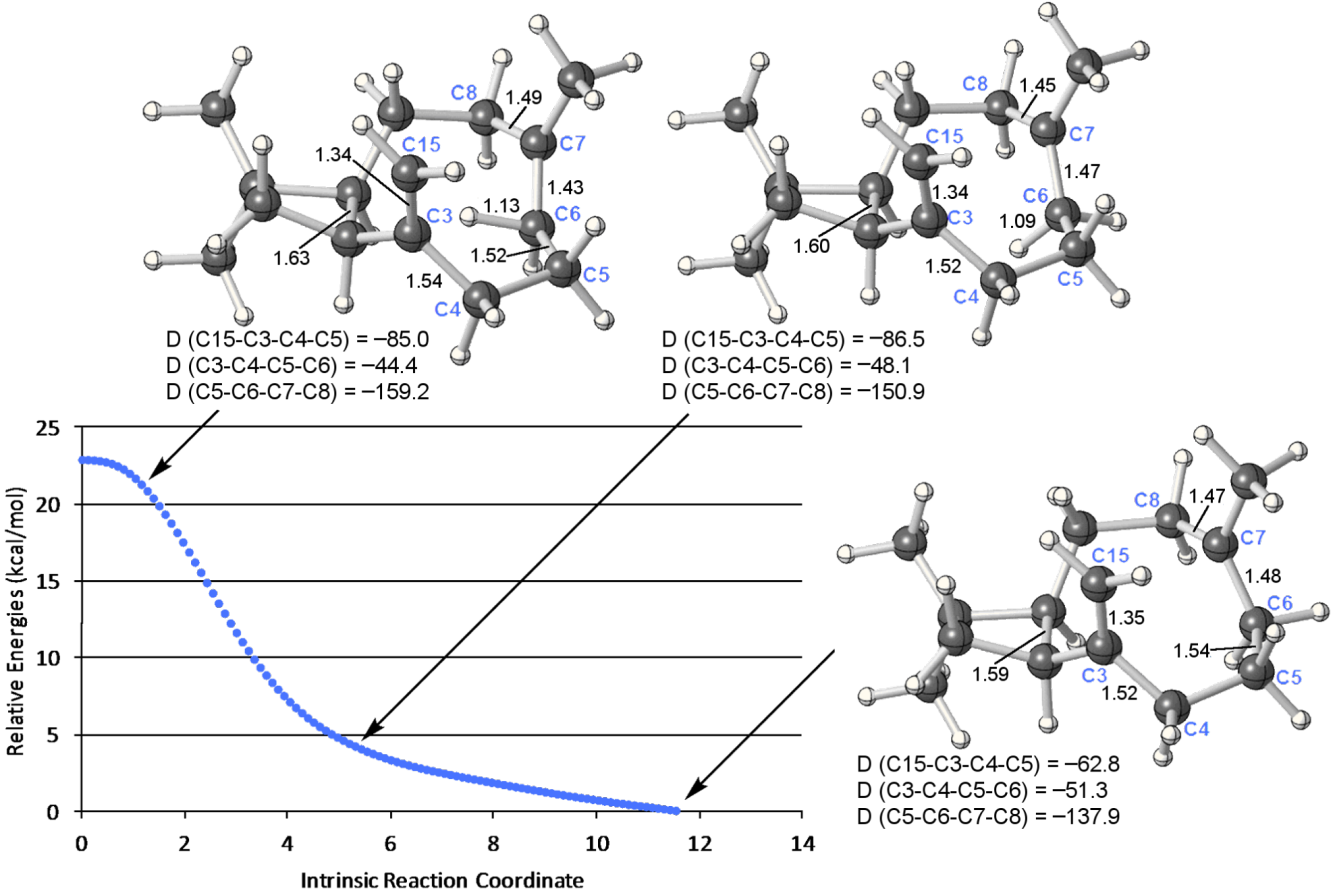
IRC from **TS-GE** toward **E**. Relative energies were calculated at the B3LYP/6-31+G(d,p) level. Selected dihedral angles are shown in degrees.

Transition-state structures for the formation of **G** from **F** and **E** from **G** were also found (Scheme 2 and Figure 5). The former, **TS-FG**, resembles a transition-state structure for a typical 1,2-hydride shift, but the predicted chemical shift of the bridging hydrogen in this structure is +4.7 ppm. The developing close contact with the C3=C15 π-bond (the H---C15 distance is only 2.32 Å in **TS-FG**) also brings this transition-state structure closer to the realm of proton sandwiches. **TS-GE** looks very similar, but with different longer and shorter H---C partial bonds and a longer H---C15 distance (2.71 Å, consistent with its lower predicted chemical shift, +3.8 ppm). The **TS-FG**/**G**/**TS-GE** energy surface is rather flat (all three structures are predicted to be within 0.5 kcal/mol of each other; Figure 5). The **G**-to-**E** reaction involves concerted but asynchronous shifting of the bridging hydrogen toward C6 and subsequent ring closure (C7–C15 bond formation). As illustrated in the IRC plot shown in Figure 6, these two events are essentially separate, with structures part way along the pathway to resembling **D**. The two events are separated by a conformational reorganization in which some C–C bonds twist and release some strain (e.g., along the C5–C6 bond) while orienting the formally empty p-orbital on C7 toward the C3=C15 π-bond. Although the IRC calculation stopped while C15 and C7 were still 3.12 Å away, optimization of the final point led to **E**. Thus, a complete pathway to **E** was found, but this pathway differs in several ways from the pathway we expected to find.

**Figure 7:**
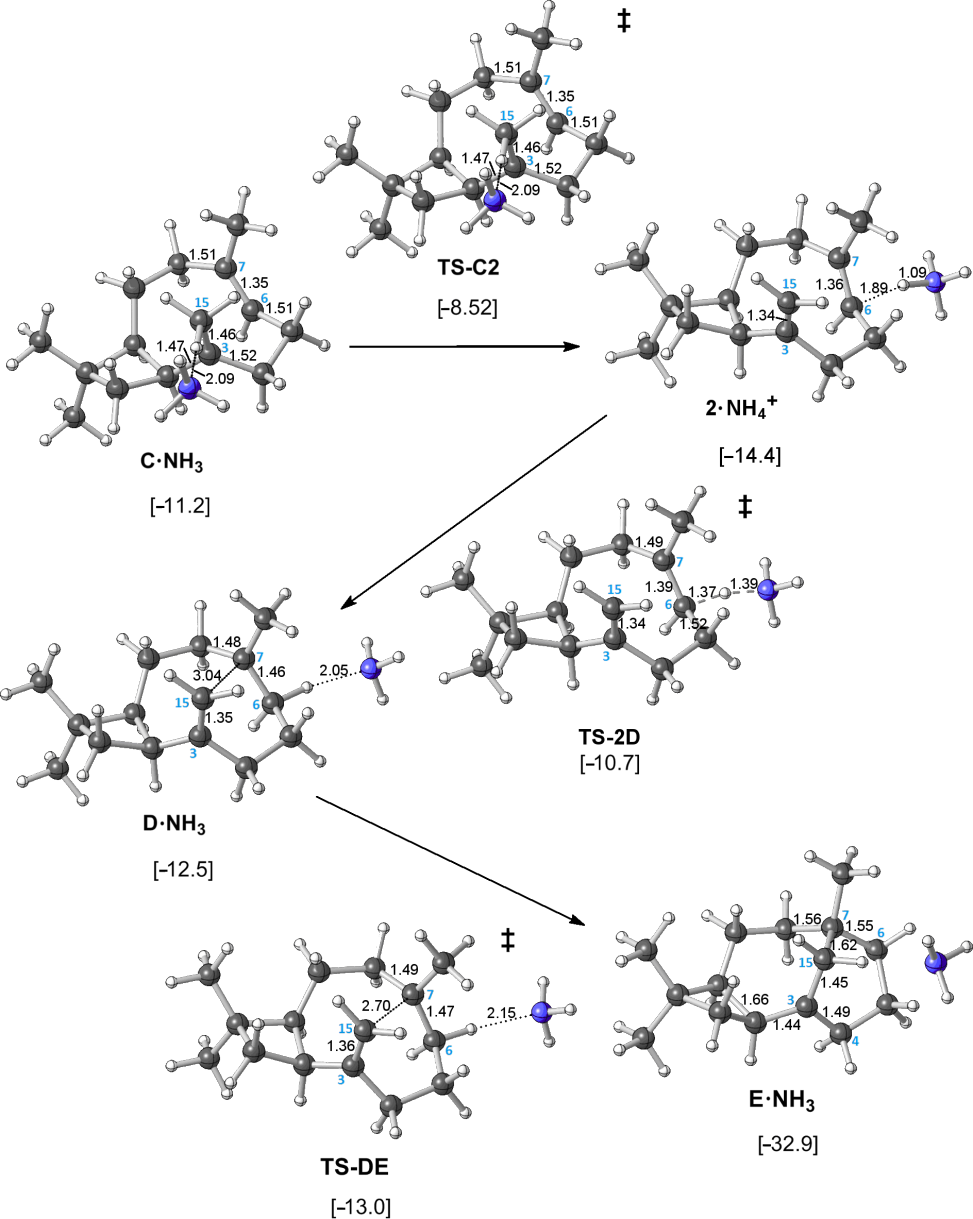
Computed pathway for the conversion of **C** to **E** in the presence of ammonia. Relative energies shown (kcal/mol) were calculated at the mPW1PW91/6-31+G(d,p)//B3LYP/6-31+G(d,p) level. Here energies are relative to that for a complex of **A** and NH_3_.

**Figure 8:**
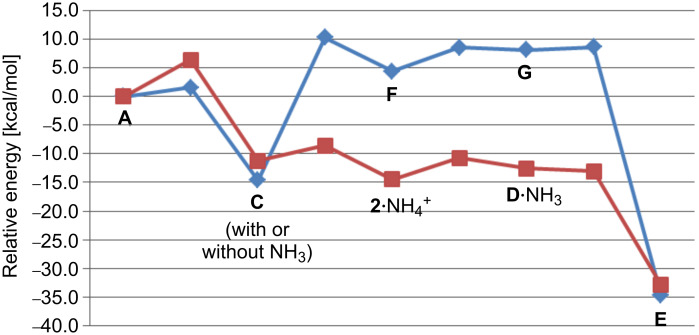
Predicted energetics for the conversion of **A** to **E** in the absence (blue) and presence (auburn) of ammonia. Relative energies shown (kcal/mol) were calculated at the mPW1PW91/6-31+G(d,p)//B3LYP/6-31+G(d,p) level. Energies for the pathway with ammonia are relative to those for an **A**·NH_3_ complex.

**Alternative mechanism:** The pathway just described represents the inherent reactivity of the carbocations involved in the formation of **E**, but how might the mechanism change if we allowed for an enzymatic base to be involved? Would the same unusual sequence of events associated with the intramolecular proton transfer persist? Would a lower energetic pathway present itself? No changes to the formation of **C** were predicted in the presence of an ammonia molecule (a simple model base [52–55]), but a stepwise proton-transfer process was found in which the C15 methyl group was first deprotonated to form an ion·molecule complex (**2**·NH_4_^+^ in Figure 7; **2** is a caryophyllene) and then C6 was protonated to form **D** (Scheme 2, right). This surprisingly elusive tertiary carbocation appears to be stabilized in the presence of a C–H hydrogen-bond acceptor that interacts with its hyperconjugated hydrogen [52–56]. Here a tertiary carbocation requires selective stabilization in order to exist as a minimum; although it is inherently lower in energy than the secondary cation **F** (on the basis of single-point calculations without ammonia present), without the intermolecular C–H···X interaction described, there is no barrier for its conversion to **E** (removal of the ammonia molecule from **D**·NH_3_ and reoptimization led to structure **E**) [57]. Still, even though tertiary carbocation **D** is predicted to exist as a minimum in the presence of a suitable base at the B3LYP/6-31+G(d,p) level, its conversion to **E** is predicted to be barrierless at the mPW1PW91/6-31+G(d,p)//B3LYP/6-31+G(d,p) level (Figure 7 and Figure 8). Thus, this ostensibly normal tertiary carbocation lacks the kinetic stability generally associated with the presence of three alkyl groups. Although cation **D** can be formed by protonation of an alkene as shown, such a scenario would likely require a separate base and acid positioned on opposite sides of the hydrocarbon substrate due to the steric congestion at its core (note the position of NH_3_ throughout Figure 7), a scenario that could be probed by deuterium labeling of farnesyl diphosphate if a suitable caryolene synthase were isolated.

The energetics for both pathways shown in Scheme 2 are summarized in Figure 8. The ammonia-free pathway has a substantial barrier, approximately 25 kcal/mol, after formation of **C**, but the ammonia-assisted pathway does not. In fact, after a small barrier for the formation of **C**, the potential energy surface for the deprotonation/reprotonation pathway is rather flat, indicating that once **C** is formed, transformation to **E** should be facile, provided that the architecture of the active site supports deprotonation/reprotonation.

## Conclusion

Which pathway to caryolene is more likely? On the basis of our computed energetics (Figure 8), we favor a mechanism for caryolene formation that involves a concerted but asynchronous [2 + 2] cycloaddition, deprotonation by an enzyme active-site base (as yet, with identity unknown [55,58–64]), and concerted but asynchronous reprotonation/cyclization (Scheme 2, right). However, several interesting structures with unusual bonding arrays are encountered along the base-free pathway.

## Experimental

All calculations were carried out with Gaussian 09 [65]. Geometry optimizations, frequency calculations and intrinsic reaction coordinate (IRC) [66–67] calculations were first carried out with B3LYP/6-31G(d) [68–72]. For IRC calculations, force constants were recalculated after every three points or at every point in the event of prematurely terminated jobs. All molecules were then subjected to optimization and frequency calculations at the B3LYP/6-31+G(d,p) level of theory [8,68–72]. Single-point energies were also calculated at the mPW1PW91/6-31+G(d,p) level [73–74] for comparison, since B3LYP energies are generally unreliable when comparing cyclic and acyclic isomers that differ in the number of σ- and π-bonds [8,74–75]. Chemical shifts (^1^H and ^13^C) in chloroform (treated with the SMD solvation model [76]) for selected structures were calculated by using mPW1PW91/6-311+G(2d,p) [11–13,73–74]. Computed scaling factors (slope = −1.0823 for ^1^H and −1.0448 for ^13^C; intercept = 31.8486 for ^1^H and 186.0596 for ^13^C) were used to convert computed isotropic values into chemical shifts [11–13].

## Supporting Information

File 1 Coordinates and energies for all computed structures, IRC plots, additional computational details and full Gaussian citation.
