# Supplementary material for: Caryolene-forming carbocation rearrangements
Source: Beilstein J Org Chem. 2013 Feb 13;9:323–31. doi: 10.3762/bjoc.9.37 (PMC3596059; doi:10.3762/bjoc.9.37)
Supplement: File 1 — Coordinates and energies for all computed structures, IRC plots, additional computational details and full Gaussian citation. [file Beilstein_J_Org_Chem-09-323-s001.pdf]

Supporting Information

for

Caryolene-forming carbocation rearrangements

Quynh Nhu N. Nguyen and Dean J. Tantillo*

Address: Department of Chemistry, University of California–Davis, 1 Shields Avenue, Davis, CA 95616, USA

Email: Dean J. Tantillo* - djtantillo@chem.ucdavis.edu

*Corresponding author

This is part 10 of our series on sesquiterpene-related calculations. For part 9 see [1]

Coordinates and energies for all computed structures, IRC plots, additional computational details and full Gaussian citation

Content

Geometry comparison of **1** and hydrogenated **1** S2

Alternative conformers of **IntB** S2

Simple model for 1,2-hydride shift S3

Chemical shift of selected atoms in several stages S3

Charges of selected atoms from NPA S3

IRC from TS-GE to E at B3LYP/6-31G(d) level S4

Calculation of energies S5–S7

Structural coordinates S8–S25

References S26

<u>Geometry comparison:</u> **1** is caryol-7-en-6-ol and **2** is hydrogenated **1**

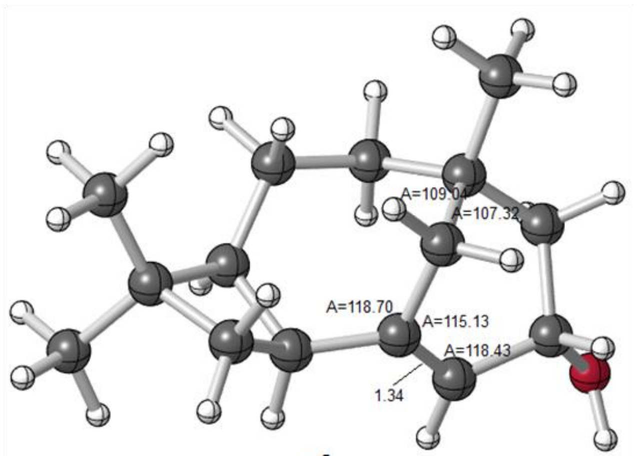

1

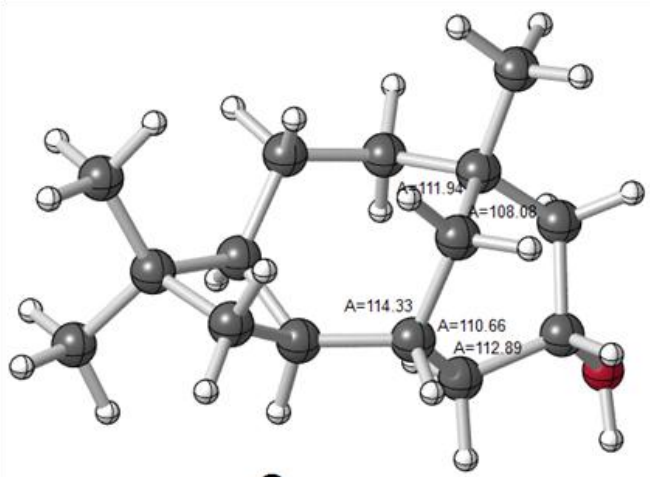

2

<u>Examining alternative conformers</u> <u>of</u> **<u>IntB</u>** <u>to derive</u> **<u>IntD'</u>**<u>-type:</u>

We selected the structure in which the methyl on C7 points up while the methyl on C3 points down, because it enabled us to arrive at the correct final carbon skeleton. However, this species proved to be unsuitable to produce the desirable product. First, one of the C15 methyl hydrogen could not be transferred to C6. Second, even after deprotonation of C15 and reprotonation of C6 by a catalyst, the C3=C16 bond failed to swing out of the ring and upward to attack the positively charged C7 in order to generate the correctly fused ring system as illustrated below.

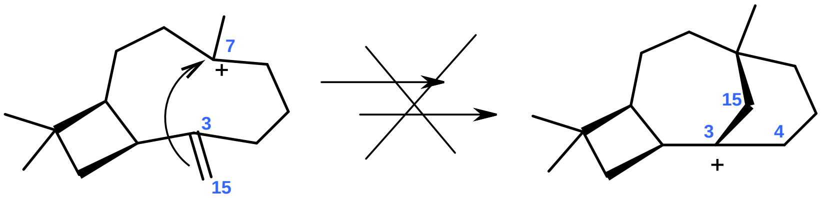

IntB
(dif. conf.)

Int E'

A simple model for 1,2-hydride shift: Model structures for a 1,2-hydride shift with predicted chemical shifts (in ppm). NMR calculations were performed at mPW1PW91/6-311+G(2d,p) on B3LYP/6-31+G(d,p) optimized structures.

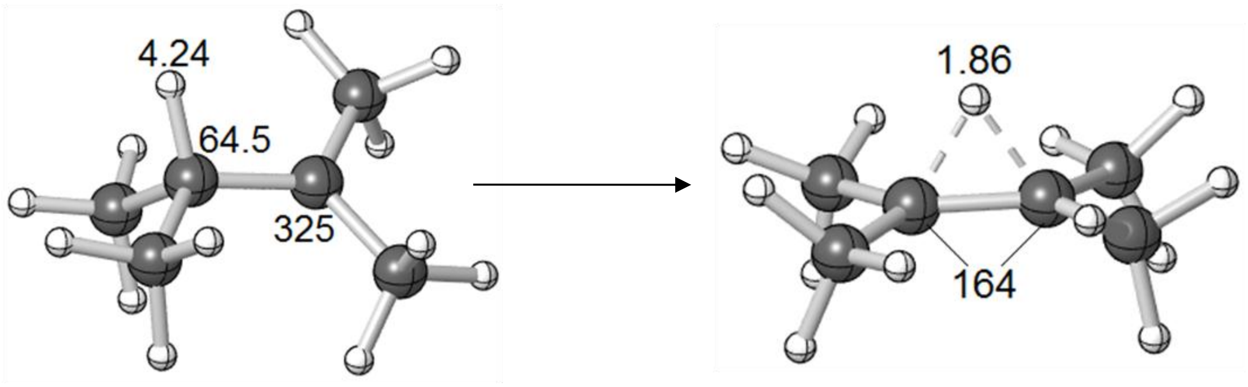

Chemical shifts of selected atoms: NMR calculated at mPW1PW91/6-311+G(2d,p) on B3LYP/6-31+G(d,p) optimized structures

Chemical shift (in ppm)							
	C	TS-CF	F	TS-FG	G	TS-GE	E
migrating H	2.64	9.15	3.76	4.65	4.05	3.82	0.98
C3	185.77	196.57	147.73	150.09	149.03	148.33	145.20
C15	29.99	110.90	122.21	131.85	129.39	126.79	35.34
C6	112.90	182.84	284.51	184.27	132.83	104.36	43.89
C7	189.99	131.14	69.75	136.83	185.87	226.42	36.91

Charges of selected atoms from Natural Population Analysis: calculated at mPW1PW91/6-31+G(d,p) on B3LYP/6-31+G(d,p) optimized structures

NPA charges						
	C	TS-CF	F	TS-FG	G	TS-GE
migrating H	0.27935	0.35162	0.30252	0.40896	0.41597	0.40981
C3	0.26422	0.1486	−0.05545	−0.08546	−0.10217	−0.10459
C15	−0.77135	−0.52768	−0.40087	−0.38897	−0.39378	−0.39749
C6	−0.26571	−0.02413	0.24904	−0.03173	−0.25225	−0.39194
C7	0.19512	−0.21496	−0.36663	−0.12836	0.10388	0.2525

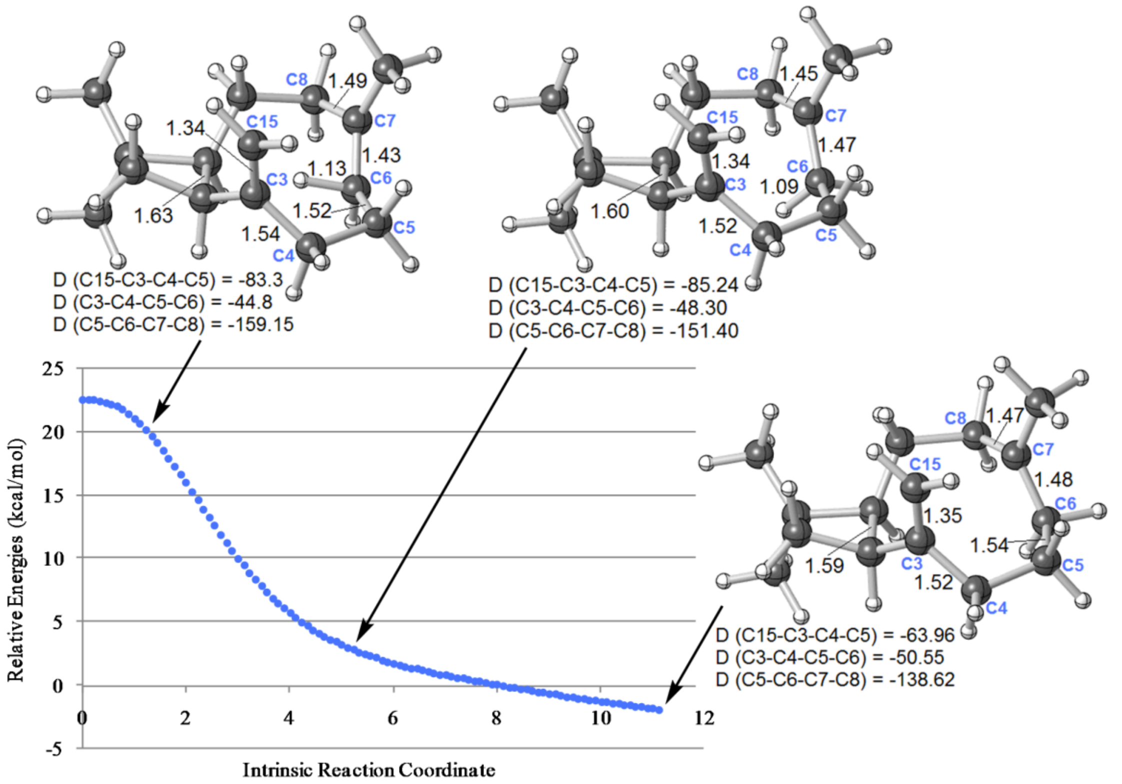

IRC from **TS-GE** toward **E** (at B3LYP/6-31G(d); the energy of **A** at this same level was set to zero). Selected dihedral angles are shown in degrees.

Calculation of energies for the two pathways to caryolene: Pathway 1 (in blue) utilizes intramolecular proton transfer and nonclassical carbocations, whereas Pathway 2 (in red) requires an enzymatic base. Zero for Pathway 1 is the energy of **IntA** alone, whereas zero for Pathway 2 is the energy of the complex of **InA** and NH_3.

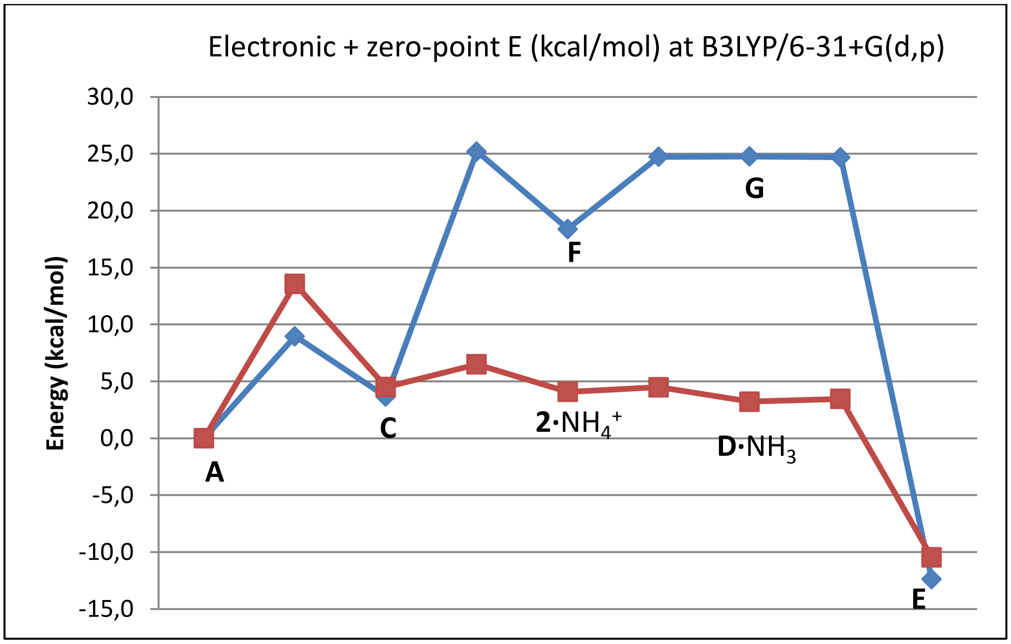

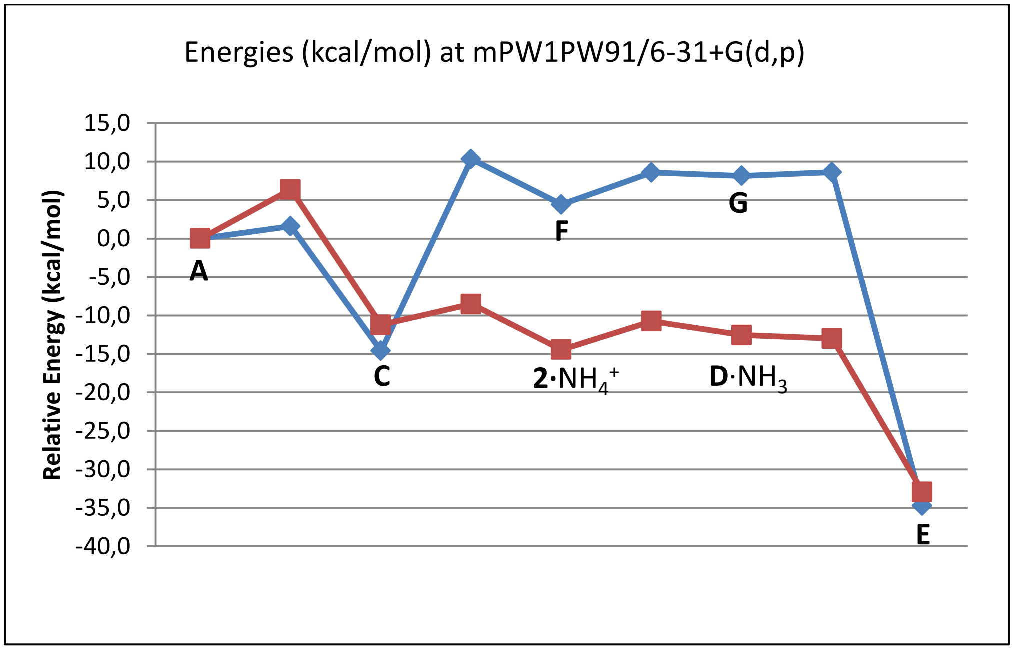

Energies calculated at B3LYP/6-31+G(d,p)						
Pathway 1				Pathway 2 (with NH_3/NH_4^+ present)		
	Electronic + zero-point energies	Rel. E (kcal/mol)			Electronic + zero-point energies	Rel. E (kcal/mol)
**A**	−586.02743	0.0		**A·NH_3**	−642.57336	0.0
**TS-AC**	−586.01314	9.0		**TS-AC**	−642.55175	13.6
**C**	−586.02159	3.7		**C·NH_3**	−642.56619	4.5
**TS-CF**	−585.98728	25.2		**TS-C2**	−642.56300	6.5
**F**	−585.99813	18.4		**2·NH_4^+**	−642.56686	4.1
**TS-FG**	−585.98802	24.7		**TS-2D**	−642.56620	4.5
**G**	−585.98797	24.8		**D·NH_3**	−642.56824	3.2
**TS-GE**	−585.98807	24.7		**TS-DE**	−642.56785	3.5
**E**	−586.04715	−12.4		**E**	−642.59002	−10.5

Energies calculated at mpw1pw91/6-31G+(d,p)						
Pathway 1				Pathway 2 (with NH_3/NH_4^+ present)		
	HF (hartrees)	Rel. E (kcal/mol)			HF (hartree)	Rel. E (kcal/mol)
**A**	−586.23890	0.0		**A·NH_3**	−642.80391	0.0
**TS-AC**	−586.23635	1.6		**TS-AC**	−642.79374	6.4
**C**	−586.26216	−14.6		**C·NH_3**	−642.82180	−11.2
**TS-CF**	−586.22245	10.3		**TS-C2**	−642.81749	−8.5
**F**	−586.23185	4.4		**2·NH_4^+**	−642.82693	−14.4
**TS-FG**	−586.22522	8.6		**TS-2D**	−642.82102	−10.7
**G**	−586.22591	8.1		**D·NH_3**	−642.82391	−12.5
**TS-GE**	−586.22516	8.6		**TS-DE**	−642.82464	−13.0
**E**	−586.29424	−34.7		**E**	−642.85639	−32.9

Energies calculated at B3LYP/6-31+G(d,p)						
Pathway 1				Pathway 2 (with NH_3/NH_4^+ present)		
	HF	Rel. E (kcal/mol)			HF	Rel. E (kcal/mol)
**A**	−586.38822	0.0		**A·NH_3**	−642.97035	0.0
**TS-AC**	−586.37572	7.8		**TS-AC**	−642.95020	12.6
**C**	−586.38846	−0.2		**C·NH_3**	−642.96857	1.1
**TS-CF**	−586.34915	24.5		**TS-C2**	−642.96274	4.8
**F**	−586.36397	15.2		**2·NH_4^+**	−642.97200	−1.0
**TS-FG**	−586.35165	22.9		**TS-2D**	−642.96637	2.5
**G**	−586.35190	22.8		**D·NH_3**	−642.97133	−0.6
**TS-GE**	−586.35170	22.9		**TS-DE**	−642.97124	−0.6
**E**	−586.41600	−17.4		**E**	−642.99550	−15.8

Structural Coordinates

****

A (minimum; frequency = 13.6)

1	C	2.477585	-1.329346	-0.088410
2	C	1.212842	-1.630860	-0.663959
3	C	-2.303111	0.376367	-0.659064
4	C	2.963246	-1.904139	1.190929
5	C	-2.098853	1.758837	-0.098425
6	C	0.496977	1.872182	0.100869
7	C	-0.769927	2.448339	-0.497248
8	H	-2.223397	1.774648	0.988247
9	H	-0.842183	3.496228	-0.173165
10	H	-0.685210	2.470507	-1.589756
11	C	-2.998749	-0.645424	-0.109074
12	C	0.198205	-2.273608	-0.020941
13	H	3.488982	-1.148057	1.783927
14	H	2.196885	-2.385509	1.797659
15	H	-2.902918	2.398676	-0.491596
16	H	-1.948718	0.241931	-1.683199
17	H	0.282418	-2.631975	1.000409
18	H	-0.748618	-2.445111	-0.518957
19	C	3.320446	-0.345233	-0.774232
20	C	1.552570	1.596070	-0.695394
21	C	2.910697	1.137702	-0.277420
22	H	3.064224	1.188404	0.802264
23	H	3.180084	-0.372377	-1.858241
24	H	1.033074	-1.263068	-1.669496
25	C	0.495754	1.724606	1.602421
26	H	0.170158	2.658786	2.075548
27	H	-0.218081	0.953577	1.916177
28	H	1.470593	1.468799	2.021248
29	C	-3.628660	-0.608999	1.261665
30	H	-4.721277	-0.544656	1.173495
31	H	-3.421049	-1.534027	1.813517
32	H	-3.298550	0.234310	1.870730
33	C	-3.305745	-1.890286	-0.908811
34	H	-3.033521	-2.805636	-0.364931
35	H	-4.385496	-1.966970	-1.090868
36	H	-2.809749	-1.892747	-1.884576
37	H	3.680279	1.763098	-0.743819
38	H	4.377886	-0.473154	-0.529566
39	H	3.719951	-2.665759	0.943921
40	H	1.434571	1.762834	-1.766420

****

TS-AC (transition state structure; imaginary frequency = −112.7)

1	C	1.079112	-1.778236	0.234504
2	C	-0.184204	-1.713109	-0.237047
3	C	-1.483077	0.747964	-0.507235
4	C	1.458186	-1.729408	1.690701
5	C	-0.948617	1.956859	0.147603
6	C	1.548556	1.620493	-0.182503
7	C	0.350675	2.502959	-0.494327
8	H	-0.844025	1.806238	1.231191
9	H	0.510407	3.520308	-0.120986
10	H	0.201073	2.586450	-1.576729
11	C	-2.531434	-0.108224	-0.101261
12	C	-1.388573	-1.342472	0.542724
13	H	2.231442	-0.980200	1.883811
14	H	0.621150	-1.541468	2.366043
15	H	-1.773023	2.695310	0.075726
16	H	-1.005591	0.480319	-1.450377
17	H	-1.177302	-1.001422	1.553835
18	H	-2.171678	-2.107035	0.551173
19	C	2.229962	-1.838192	-0.744808
20	C	1.933251	0.669447	-1.056174
21	C	2.932093	-0.438881	-0.845221
22	H	3.530518	-0.266323	0.052302
23	H	1.864107	-2.128317	-1.735451
24	H	-0.338228	-1.891974	-1.300126
25	C	2.187595	1.888979	1.158133
26	H	2.546975	2.924740	1.196566
27	H	1.470077	1.781614	1.983673
28	H	3.038034	1.240838	1.370690
29	C	-3.415392	0.301045	1.069944
30	H	-4.163366	1.026004	0.731018
31	H	-3.954954	-0.565966	1.460513
32	H	-2.854012	0.750831	1.892587
33	C	-3.294038	-0.793490	-1.243628
34	H	-3.880458	-1.631872	-0.857872
35	H	-3.989579	-0.082160	-1.699451
36	H	-2.627724	-1.167020	-2.025831
37	H	3.636245	-0.480561	-1.684353
38	H	2.978451	-2.579704	-0.443051
39	H	1.888510	-2.697965	1.975642
40	H	1.418387	0.634932	-2.018966

****

C (minimum; frequency = 48.2)

1	C	0.818664	-1.110363	0.332594
2	C	-0.568386	-0.987937	-0.212902
3	C	-1.201545	0.416565	-0.616405
4	C	1.067995	-0.784819	1.776548
5	C	-0.591689	1.695243	-0.046210
6	C	1.902520	1.197597	-0.347192
7	C	0.761331	2.121171	-0.689428
8	H	-0.493345	1.656243	1.043991
9	H	1.001254	3.134837	-0.355686
10	H	0.630066	2.155832	-1.777802
11	C	-2.568936	-0.076367	0.000220
12	C	-1.794248	-1.210792	0.735333
13	H	2.130152	-0.718425	2.018729
14	H	0.549145	0.103261	2.136799
15	H	-1.291806	2.512369	-0.251155
16	H	-1.244710	0.523621	-1.707871
17	H	-1.579284	-0.945016	1.773418
18	H	-2.227234	-2.215251	0.713290
19	C	1.797120	-2.160228	-0.228895
20	C	1.976925	-0.026205	-0.982663
21	C	2.900276	-1.206534	-0.732795
22	H	3.648871	-1.005260	0.035355
23	H	1.337524	-2.707130	-1.056251
24	H	-0.645811	-1.644646	-1.083111
25	C	2.864786	1.665491	0.692636
26	H	3.380340	2.550841	0.294183
27	H	2.342100	2.008956	1.593967
28	H	3.622276	0.931854	0.967069
29	C	-3.338895	0.885047	0.907857
30	H	-3.757450	1.724159	0.341202
31	H	-4.177904	0.358911	1.376247
32	H	-2.720493	1.293221	1.713558
33	C	-3.496735	-0.621707	-1.094660
34	H	-4.354698	-1.137613	-0.650330
35	H	-3.882290	0.193088	-1.717410
36	H	-2.987912	-1.333788	-1.754060
37	H	3.415493	-1.550032	-1.632785
38	H	2.114785	-2.878288	0.534335
39	H	0.674093	-1.644459	2.339547
40	H	1.341747	-0.144273	-1.859459

****

TS-CF (transition state structure; imaginary frequency = −837.5)

1	C	0.611968	-1.430332	0.620750
2	C	-0.726972	-1.133819	0.021948
3	C	-1.126981	0.330848	-0.555793
4	C	1.054101	-0.787542	1.749784
5	C	-0.498487	1.617105	0.005941
6	C	2.001711	1.114419	-0.288914
7	C	0.853782	2.078939	-0.597994
8	H	-0.410508	1.577156	1.098685
9	H	1.087637	3.072558	-0.204381
10	H	0.745710	2.188325	-1.683720
11	C	-2.595662	0.022154	-0.039041
12	C	-2.005993	-1.073407	0.894620
13	H	1.456919	0.174730	0.580401
14	H	0.378287	-0.215817	2.376793
15	H	-1.218502	2.419598	-0.189708
16	H	-1.056831	0.356905	-1.649572
17	H	-1.804979	-0.678724	1.895627
18	H	-2.560486	-2.011176	0.994646
19	C	1.613620	-2.188146	-0.202195
20	C	2.159839	0.035945	-1.148556
21	C	2.795073	-1.243460	-0.797798
22	H	3.583122	-1.145085	-0.049642
23	H	1.125454	-2.693706	-1.036488
24	H	-0.888137	-1.834747	-0.799345
25	C	3.095455	1.614457	0.653424
26	H	3.609192	2.446204	0.158967
27	H	2.671639	1.998682	1.585381
28	H	3.840493	0.853191	0.892515
29	C	-3.367828	1.139003	0.665826
30	H	-3.653323	1.936740	-0.028980
31	H	-4.292872	0.730625	1.087570
32	H	-2.805770	1.587652	1.490525
33	C	-3.457280	-0.572496	-1.163449
34	H	-4.397948	-0.961785	-0.759765
35	H	-3.704980	0.193170	-1.906942
36	H	-2.958349	-1.395310	-1.687531
37	H	3.191638	-1.770131	-1.668258
38	H	2.127154	-2.937422	0.407776
39	H	2.027909	-1.015018	2.176967
40	H	1.469457	-0.018620	-1.992847

****

F (minimum; frequency = 41.8)

1	C	-0.304460	1.780706	0.346654
2	C	0.887700	1.062381	-0.237761
3	C	1.034790	-0.522046	-0.401298
4	C	-0.385822	2.190492	1.623409
5	C	0.357226	-1.451677	0.612782
6	C	-2.190236	-1.072144	0.280112
7	C	-1.002456	-2.046190	0.202370
8	H	0.268791	-0.973722	1.596818
9	H	-1.237539	-2.879909	0.873306
10	H	-0.937498	-2.470850	-0.808257
11	C	2.591406	-0.301658	-0.165728
12	C	2.254741	1.075119	0.477116
13	H	-2.261087	-0.595483	1.263438
14	H	0.401771	1.994504	2.342661
15	H	1.014927	-2.314678	0.760506
16	H	0.811315	-0.881430	-1.415564
17	H	2.158813	0.979640	1.563494
18	H	2.915575	1.920054	0.259388
19	C	-1.406727	2.145680	-0.621710
20	C	-2.332710	-0.101266	-0.775223
21	C	-2.771491	1.251888	-0.574609
22	H	-3.241727	1.409123	0.397314
23	H	-1.049495	2.141171	-1.654225
24	H	1.032565	1.475045	-1.242646
25	C	-3.569720	-1.858394	0.043333
26	H	-3.573451	-2.388972	-0.910998
27	H	-3.604447	-2.590042	0.856239
28	H	-4.447821	-1.214214	0.116239
29	C	3.341268	-1.283830	0.735614
30	H	3.416807	-2.279089	0.281791
31	H	4.363450	-0.922653	0.895153
32	H	2.878503	-1.389762	1.721628
33	C	3.332652	-0.160666	-1.503596
34	H	4.355466	0.195398	-1.339345
35	H	3.394689	-1.127282	-2.016483
36	H	2.843961	0.546144	-2.183126
37	H	-3.387408	1.633819	-1.393061
38	H	-1.804472	3.142130	-0.408056
39	H	-1.235903	2.762279	1.987816
40	H	-2.092738	-0.421595	-1.792812

****

TS-FG (transition state structure; imaginary frequency = −195.4)

1	C	0.667777	-1.497829	0.519553
2	C	-0.674499	-1.097278	-0.077181
3	C	-1.104706	0.389476	-0.529997
4	C	0.961670	-1.345011	1.827938
5	C	-0.506864	1.641891	0.140573
6	C	1.976644	1.267188	-0.247669
7	C	0.798671	2.206342	-0.488497
8	H	-0.350995	1.484271	1.216003
9	H	1.022206	3.180439	-0.045743
10	H	0.651251	2.363499	-1.562887
11	C	-2.568974	0.010772	-0.050679
12	C	-1.961943	-1.158924	0.776102
13	H	1.410017	0.207041	0.158151
14	H	0.253043	-0.925332	2.534495
15	H	-1.252716	2.439508	0.065870
16	H	-1.048287	0.525958	-1.618262
17	H	-1.792476	-0.857176	1.814693
18	H	-2.496241	-2.114028	0.778382
19	C	1.712348	-2.115360	-0.412501
20	C	2.128300	0.183996	-1.121177
21	C	2.840629	-1.092817	-0.877927
22	H	3.616292	-1.004430	-0.115447
23	H	1.224197	-2.519419	-1.303335
24	H	-0.821026	-1.723142	-0.962385
25	C	2.971218	1.639643	0.828533
26	H	3.517005	2.522286	0.475705
27	H	2.457596	1.921345	1.751642
28	H	3.695544	0.854469	1.046733
29	C	-3.361938	1.040657	0.756510
30	H	-3.652372	1.903546	0.146053
31	H	-4.285364	0.580119	1.124541
32	H	-2.813625	1.406983	1.630104
33	C	-3.418130	-0.486126	-1.230803
34	H	-4.351346	-0.931137	-0.869479
35	H	-3.680111	0.342820	-1.898033
36	H	-2.902240	-1.245268	-1.828990
37	H	3.294026	-1.465133	-1.800498
38	H	2.226469	-2.942526	0.084566
39	H	1.913399	-1.672345	2.239265
40	H	1.404534	0.147937	-1.937802

****

G (minimum; frequency = 58.6)

1	C	0.671768	-1.559873	0.474032
2	C	-0.662373	-1.109842	-0.118510
3	C	-1.082135	0.399621	-0.516058
4	C	0.931978	-1.508941	1.794860
5	C	-0.483439	1.637775	0.185715
6	C	1.986992	1.359036	-0.257835
7	C	0.791774	2.264321	-0.468617
8	H	-0.287376	1.444348	1.248431
9	H	0.990853	3.236591	-0.011048
10	H	0.613416	2.424659	-1.537094
11	C	-2.549656	0.015038	-0.047249
12	C	-1.955616	-1.198804	0.721772
13	H	1.345243	0.149349	-0.024657
14	H	0.212338	-1.132503	2.514639
15	H	-1.241341	2.426395	0.163668
16	H	-1.032125	0.569365	-1.599709
17	H	-1.789447	-0.950268	1.774739
18	H	-2.498963	-2.147629	0.674785
19	C	1.739958	-2.125588	-0.476728
20	C	2.080984	0.221887	-1.068583
21	C	2.835389	-1.059326	-0.856117
22	H	3.575921	-0.972057	-0.058540
23	H	1.263794	-2.486249	-1.393880
24	H	-0.806138	-1.692830	-1.033491
25	C	2.911102	1.660780	0.880102
26	H	3.492339	2.542666	0.578676
27	H	2.354827	1.941416	1.779326
28	H	3.609283	0.856835	1.110620
29	C	-3.327908	1.013782	0.812023
30	H	-3.616255	1.906475	0.244776
31	H	-4.252385	0.543576	1.164685
32	H	-2.771294	1.334255	1.698526
33	C	-3.407539	-0.413469	-1.247650
34	H	-4.345842	-0.862758	-0.905241
35	H	-3.659930	0.449201	-1.874663
36	H	-2.902074	-1.150422	-1.881196
37	H	3.353972	-1.353505	-1.772718
38	H	2.242908	-2.980902	-0.017828
39	H	1.868350	-1.880644	2.203705
40	H	1.392253	0.206264	-1.913466

****

TS-GE (transition state structure; imaginary frequency = −195.6)

1	C	0.696899	-1.605412	0.436570
2	C	-0.634432	-1.114420	-0.139195
3	C	-1.062294	0.410138	-0.505371
4	C	0.927301	-1.674374	1.759857
5	C	-0.489653	1.648062	0.219587
6	C	1.957689	1.425587	-0.231773
7	C	0.771373	2.323586	-0.436761
8	H	-0.283465	1.440824	1.277120
9	H	0.948654	3.289774	0.042103
10	H	0.578211	2.491580	-1.500790
11	C	-2.528829	0.000119	-0.048648
12	C	-1.927618	-1.224895	0.694953
13	H	1.205255	0.119339	-0.247278
14	H	0.194029	-1.363713	2.496885
15	H	-1.260739	2.423627	0.210715
16	H	-1.011248	0.599886	-1.585271
17	H	-1.764013	-0.993759	1.752203
18	H	-2.465373	-2.175904	0.630350
19	C	1.783538	-2.091133	-0.540301
20	C	2.064038	0.294813	-1.067675
21	C	2.847011	-0.982353	-0.855994
22	H	3.551655	-0.893764	-0.026371
23	H	1.316615	-2.415153	-1.476856
24	H	-0.779763	-1.664446	-1.074539
25	C	2.798074	1.643299	0.975926
26	H	2.204933	1.983598	1.829105
27	H	3.411542	0.788275	1.255199
28	H	3.473487	2.472498	0.712706
29	C	-3.319159	0.971496	0.830761
30	H	-3.617301	1.873198	0.283170
31	H	-4.238600	0.482590	1.170777
32	H	-2.767722	1.277503	1.725554
33	C	-3.377959	-0.405655	-1.263027
34	H	-4.313980	-0.870077	-0.934777
35	H	-3.635066	0.469891	-1.869937
36	H	-2.864592	-1.123322	-1.911971
37	H	3.418462	-1.235060	-1.753196
38	H	2.301146	-2.960490	-0.126416
39	H	1.854196	-2.085881	2.151684
40	H	1.507804	0.355844	-2.003615

****

E (minimum; frequency = 54.3)

1	C	0.574351	-1.099540	0.482251
2	C	-0.835102	-1.193916	0.222440
3	C	-1.348881	0.055646	-0.768353
4	C	1.175942	0.015634	1.199902
5	C	-0.523229	1.338787	-0.776103
6	C	1.924251	0.835689	0.030204
7	C	0.956731	1.127520	-1.158630
8	H	-0.600968	1.875355	0.174099
9	H	1.336713	2.017740	-1.669675
10	H	1.014582	0.320931	-1.900799
11	C	-2.691479	0.024758	0.050025
12	C	-1.959832	-0.740493	1.189531
13	H	3.274633	0.151927	-1.523171
14	H	0.464368	0.640773	1.735988
15	H	-0.996848	1.993655	-1.517233
16	H	-1.415315	-0.340918	-1.786901
17	H	-1.594532	-0.071337	1.972786
18	H	-2.499838	-1.569242	1.656160
19	C	1.524890	-2.016668	-0.199606
20	C	3.131713	-0.018781	-0.451046
21	C	2.984161	-1.535479	-0.164217
22	H	3.401073	-1.758636	0.823851
23	H	1.153573	-2.217491	-1.215352
24	H	-1.081590	-2.124799	-0.291758
25	C	2.387719	2.160204	0.662866
26	H	1.542493	2.796341	0.944653
27	H	2.996806	1.986119	1.555307
28	H	2.997151	2.714131	-0.059339
29	C	-3.315398	1.366055	0.438614
30	H	-3.681883	1.907808	-0.439902
31	H	-4.174314	1.190311	1.095420
32	H	-2.622605	2.017596	0.978389
33	C	-3.734526	-0.854362	-0.656589
34	H	-4.592925	-1.027541	0.000674
35	H	-4.102072	-0.364914	-1.564736
36	H	-3.334991	-1.833564	-0.942904
37	H	3.575083	-2.120333	-0.873780
38	H	1.427702	-2.991346	0.311929
39	H	1.959747	-0.313637	1.889927
40	H	4.046090	0.330750	0.038641

****

A·NH_3 (minimum; frequency = 15.5)

1	C	-2.289950	-0.079965	0.054257
2	C	-1.178976	0.825560	0.134187
3	C	2.787031	0.526695	-0.732907
4	C	-3.445664	-0.027748	0.950161
5	C	3.329405	-0.696875	-0.037459
6	C	1.153763	-2.101755	0.109822
7	C	2.579458	-2.006002	-0.393598
8	H	3.360332	-0.560954	1.048202
9	H	3.150033	-2.844957	0.027105
10	H	2.593315	-2.134972	-1.481804
11	C	2.759080	1.795367	-0.282705
12	C	-1.122386	1.887484	0.974605
13	H	-3.873225	-1.022518	1.111796
14	H	-3.273424	0.473336	1.902742
15	H	4.374945	-0.842427	-0.345238
16	H	2.457161	0.356236	-1.760045
17	H	-1.924232	2.142668	1.660720
18	H	-0.257521	2.543278	0.968251
19	C	-2.254466	-1.122912	-0.981747
20	C	0.131612	-2.150927	-0.771236
21	C	-1.322845	-2.341781	-0.490059
22	H	-1.533431	-2.559743	0.559287
23	H	-1.826421	-0.752102	-1.917844
24	H	-0.342493	0.645577	-0.535914
25	C	0.998310	-2.195342	1.606787
26	H	1.482639	-3.107588	1.977469
27	H	1.498083	-1.356749	2.104020
28	H	-0.040130	-2.213531	1.943331
29	C	3.247267	2.230313	1.079166
30	H	4.167250	2.821416	0.981899
31	H	2.516681	2.885376	1.571891
32	H	3.462792	1.396287	1.749659
33	C	2.297607	2.924773	-1.176160
34	H	1.487644	3.505586	-0.712392
35	H	3.113543	3.637328	-1.353330
36	H	1.951208	2.565645	-2.149662
37	H	-1.702019	-3.190005	-1.071606
38	H	-3.255462	-1.519353	-1.164237
39	H	-4.256938	0.542368	0.406956
40	H	0.379421	-2.095006	-1.831638
41	N	-5.876930	1.536255	-0.358882
42	H	-5.703458	2.137660	-1.161830
43	H	-6.289161	2.122135	0.364701
44	H	-6.594102	0.869921	-0.638994

****

TS-AC·NH_3 (transition state structure; imaginary frequency = –120.0)

1	C	-1.427909	-0.647354	0.375518
2	C	-0.288038	-1.272060	0.733218
3	C	2.127322	-0.015602	0.267045
4	C	-2.016904	-0.641945	-1.009247
5	C	2.313316	1.137814	-0.617526
6	C	0.206436	2.421195	-0.080356
7	C	1.727027	2.459974	-0.069707
8	H	1.956673	0.909587	-1.632896
9	H	2.103920	3.284511	-0.684251
10	H	2.106422	2.623505	0.945139
11	C	2.353859	-1.404977	0.009835
12	C	0.722212	-1.862722	-0.198877
13	H	-2.129769	0.377523	-1.393654
14	H	-1.440839	-1.217556	-1.737594
15	H	3.415337	1.190640	-0.757447
16	H	1.772223	0.217555	1.271286
17	H	0.496825	-1.651700	-1.243307
18	H	0.867737	-2.942507	-0.069967
19	C	-2.165597	0.183670	1.403190
20	C	-0.467277	2.040652	1.023972
21	C	-1.931383	1.713889	1.156519
22	H	-2.488709	2.029659	0.271576
23	H	-1.819817	-0.071871	2.411058
24	H	-0.034064	-1.298568	1.792340
25	C	-0.415192	2.778098	-1.407242
26	H	-0.157180	3.811253	-1.671953
27	H	-0.031209	2.147389	-2.221535
28	H	-1.502237	2.699842	-1.410912
29	C	3.105875	-1.774838	-1.269314
30	H	4.169702	-1.533881	-1.170856
31	H	3.028277	-2.850520	-1.448225
32	H	2.717317	-1.261189	-2.152793
33	C	2.879071	-2.161241	1.251025
34	H	2.848693	-3.237155	1.059077
35	H	3.917761	-1.884154	1.453548
36	H	2.285803	-1.954759	2.145800
37	H	-2.361516	2.257580	2.006206
38	H	-3.243528	-0.008535	1.365686
39	H	-3.027526	-1.074767	-0.964177
40	H	0.114426	1.879141	1.934698
41	N	-5.285293	-1.970062	-0.744498
42	H	-5.998493	-1.282296	-0.976863
43	H	-5.564953	-2.396844	0.135964
44	H	-5.341119	-2.698775	-1.452775

****

C·NH_3 (minimum; frequency = 31.1)

1	C	0.790925	0.819950	0.468841
2	C	-0.581074	0.429775	0.832126
3	C	-1.265935	-0.962202	0.341748
4	C	1.105652	1.338094	-0.860174
5	C	-0.698137	-1.751196	-0.838229
6	C	1.778278	-1.803734	-0.293342
7	C	0.535932	-2.636900	-0.518574
8	H	-0.488066	-1.106425	-1.699405
9	H	0.691857	-3.330771	-1.351138
10	H	0.315028	-3.243524	0.367592
11	C	-2.603002	-0.156445	0.111788
12	C	-1.792151	1.167194	0.168429
13	H	2.168542	1.291774	-1.104730
14	H	0.492020	0.940819	-1.669022
15	H	-1.499480	-2.424624	-1.165046
16	H	-1.323778	-1.637473	1.203478
17	H	-1.554597	1.544742	-0.829026
18	H	-2.204564	1.985427	0.766614
19	C	1.856468	1.022852	1.535344
20	C	1.988560	-1.276324	0.937440
21	C	2.883624	-0.119526	1.323455
22	H	3.592187	0.138263	0.533919
23	H	1.409021	0.950109	2.530136
24	H	-0.670730	0.447197	1.920347
25	C	2.652620	-1.578496	-1.494475
26	H	3.059629	-2.544001	-1.820731
27	H	2.077717	-1.189588	-2.344431
28	H	3.497078	-0.912802	-1.309609
29	C	-3.366997	-0.392024	-1.192415
30	H	-3.831653	-1.383668	-1.215071
31	H	-4.172325	0.345360	-1.284229
32	H	-2.730199	-0.294656	-2.077187
33	C	-3.549498	-0.310266	1.310928
34	H	-4.386216	0.392305	1.232304
35	H	-3.966034	-1.322752	1.349674
36	H	-3.045641	-0.120408	2.265501
37	H	3.456453	-0.304600	2.236838
38	H	2.306093	2.018207	1.429053
39	H	0.868137	2.440836	-0.790895
40	H	1.333869	-1.617278	1.740287
41	N	0.630012	4.505828	-0.971549
42	H	-0.299140	4.851057	-0.738570
43	H	0.792671	4.745542	-1.947930
44	H	1.290441	5.055627	-0.425208

****

TS-C2·NH_3 (transition state structure; imaginary frequency = –934.5)

1	C	0.766793	1.047721	0.318109
2	C	-0.603339	0.571202	0.668764
3	C	-1.160099	-0.926098	0.388183
4	C	1.071127	1.553350	-0.944332
5	C	-0.601423	-1.799943	-0.743060
6	C	1.858399	-1.805769	-0.206930
7	C	0.630327	-2.670164	-0.376977
8	H	-0.387217	-1.206677	-1.641325
9	H	0.778882	-3.414643	-1.167412
10	H	0.412351	-3.221265	0.545482
11	C	-2.569897	-0.257943	0.122654
12	C	-1.853412	1.106995	-0.077879
13	H	2.124806	1.694103	-1.190494
14	H	0.439557	1.270751	-1.786293
15	H	-1.408814	-2.484427	-1.028712
16	H	-1.134752	-1.510261	1.314759
17	H	-1.662560	1.292547	-1.139834
18	H	-2.330890	1.992332	0.358617
19	C	1.820551	1.151116	1.410615
20	C	2.086550	-1.240918	0.992545
21	C	2.875491	0.011656	1.262712
22	H	3.563235	0.247511	0.447252
23	H	1.341122	1.074463	2.390878
24	H	-0.732154	0.722104	1.744068
25	C	2.656542	-1.551772	-1.460193
26	H	3.066563	-2.501472	-1.825550
27	H	2.027766	-1.158337	-2.269574
28	H	3.496418	-0.869585	-1.315777
29	C	-3.390348	-0.743595	-1.074168
30	H	-3.770251	-1.759262	-0.918592
31	H	-4.259447	-0.090135	-1.212006
32	H	-2.819390	-0.734791	-2.007624
33	C	-3.438082	-0.278601	1.389745
34	H	-4.327875	0.347025	1.258016
35	H	-3.776237	-1.297292	1.608410
36	H	-2.901591	0.088457	2.271873
37	H	3.465865	-0.042240	2.183490
38	H	2.314931	2.130162	1.356933
39	H	0.689134	2.852363	-0.802611
40	H	1.467259	-1.570860	1.828036
41	N	0.368503	4.209529	-0.893545
42	H	0.873280	4.762306	-0.200350
43	H	-0.631529	4.348803	-0.746982
44	H	0.606940	4.568197	-1.818595

****

2·NH_4^+ (minimum; frequency = 32.6)

1	C	0.106612	1.832239	0.201353
2	C	1.213929	0.982297	-0.394579
3	C	1.299310	-0.614169	-0.404703
4	C	0.126892	2.271709	1.468786
5	C	0.648836	-1.443851	0.711788
6	C	-1.759718	-0.758412	0.500354
7	C	-0.806957	-1.924344	0.430766
8	H	0.681503	-0.914075	1.671393
9	H	-1.071531	-2.693825	1.165510
10	H	-0.826292	-2.390400	-0.562547
11	C	2.871125	-0.447934	-0.284389
12	C	2.638329	1.009354	0.206199
13	H	1.244127	-2.353180	0.848450
14	H	0.998897	-1.047462	-1.368923
15	H	2.630049	1.054524	1.299890
16	H	3.313317	1.787861	-0.164672
17	C	-1.015782	2.283590	-0.736133
18	C	-1.970697	-0.002598	-0.610670
19	C	-2.336678	1.459272	-0.619831
20	H	-2.860244	1.747296	0.296121
21	H	-0.664948	2.228221	-1.773145
22	H	1.292671	1.278669	-1.446635
23	C	-2.296493	-0.434011	1.871233
24	H	-2.824391	-1.302368	2.287291
25	H	-1.470567	-0.221338	2.558395
26	H	-2.965976	0.427496	1.890126
27	C	3.633478	-1.354049	0.685006
28	H	3.647803	-2.396858	0.345353
29	H	4.675540	-1.021429	0.754446
30	H	3.217700	-1.330193	1.697369
31	C	3.536972	-0.500161	-1.667979
32	H	4.584003	-0.183480	-1.603977
33	H	3.520980	-1.520104	-2.070385
34	H	3.039291	0.152443	-2.393716
35	H	-2.984947	1.726111	-1.465303
36	H	-1.259802	3.334310	-0.545498
37	H	-1.489114	-0.354080	-1.527925
38	H	-0.643085	2.938174	1.850470
39	H	0.918452	2.011105	2.163815
40	N	-4.553136	-1.375544	-1.096457
41	H	-4.711598	-1.173992	-2.087413
42	H	-5.296784	-0.941100	-0.543427
43	H	-4.575259	-2.388715	-0.952345
44	H	-3.582787	-0.978393	-0.790725

****

TS-2D·NH_3 (transition state structure; imaginary frequency = –873.5)

1	C	0.036472	1.750725	0.283187
2	C	1.189205	1.015243	-0.369743
3	C	1.340040	-0.570003	-0.446679
4	C	0.018972	2.072594	1.586842
5	C	0.732785	-1.439964	0.657682
6	C	-1.705307	-0.850565	0.543046
7	C	-0.723443	-1.963201	0.398639
8	H	0.769394	-0.938039	1.631291
9	H	-0.931432	-2.762422	1.117988
10	H	-0.756223	-2.385223	-0.612165
11	C	2.905365	-0.347014	-0.325680
12	C	2.614974	1.078100	0.226715
13	H	1.339340	-2.345975	0.755783
14	H	1.047116	-0.988396	-1.419257
15	H	2.612654	1.079503	1.321200
16	H	3.253475	1.898440	-0.116552
17	C	-1.104724	2.207130	-0.624147
18	C	-2.079617	-0.105154	-0.573041
19	C	-2.412526	1.380227	-0.492044
20	H	-2.910347	1.620337	0.451529
21	H	-0.776700	2.172276	-1.669127
22	H	1.242179	1.369106	-1.404659
23	C	-2.284117	-0.650239	1.907232
24	H	-2.917029	-1.517419	2.147267
25	H	-1.488720	-0.647545	2.659110
26	H	-2.873599	0.259027	2.016234
27	C	3.708027	-1.264074	0.599821
28	H	3.757456	-2.290293	0.216005
29	H	4.737779	-0.896961	0.675481
30	H	3.302383	-1.297620	1.616175
31	C	3.560684	-0.315127	-1.714680
32	H	4.594678	0.039914	-1.643585
33	H	3.581783	-1.317286	-2.158744
34	H	3.032306	0.346761	-2.409556
35	H	-3.097522	1.673067	-1.296992
36	H	-1.350341	3.253875	-0.411944
37	H	-1.527487	-0.369428	-1.481661
38	H	-0.792078	2.656307	2.016107
39	H	0.821826	1.803303	2.265524
40	N	-4.520894	-1.136583	-1.299885
41	H	-4.632712	-2.143915	-1.182712
42	H	-4.611748	-0.922547	-2.293298
43	H	-5.282621	-0.671285	-0.805869
44	H	-3.278296	-0.713536	-0.827277

****

D·NH_3 (minimum; frequency = 26.8)

1	C	0.076331	1.551019	0.448720
2	C	1.304990	1.046644	-0.265598
3	C	1.494863	-0.498226	-0.580119
4	C	-0.096705	1.417618	1.781639
5	C	0.830605	-1.497910	0.359839
6	C	-1.659474	-0.798067	0.407576
7	C	-0.673492	-1.848621	0.053807
8	H	0.912538	-1.195936	1.408345
9	H	-0.901948	-2.753581	0.628081
10	H	-0.747815	-2.076725	-1.015180
11	C	3.045112	-0.287474	-0.325469
12	C	2.695636	1.035984	0.417899
13	H	1.352320	-2.455208	0.266599
14	H	1.257215	-0.774086	-1.615540
15	H	2.632647	0.881686	1.499569
16	H	3.334202	1.904902	0.231694
17	C	-0.989554	2.245783	-0.384076
18	C	-2.217543	0.061373	-0.630651
19	C	-2.370073	1.562890	-0.314640
20	H	-2.830076	1.703730	0.667737
21	H	-0.668932	2.291413	-1.429989
22	H	1.383268	1.583045	-1.215083
23	C	-2.315895	-0.899482	1.737448
24	H	-3.059276	-1.709367	1.633404
25	H	-1.621967	-1.220186	2.517711
26	H	-2.849943	-0.001165	2.043238
27	C	3.800539	-1.335335	0.494793
28	H	3.886188	-2.287532	-0.041991
29	H	4.818723	-0.982124	0.692314
30	H	3.332952	-1.530165	1.465356
31	C	3.780927	-0.035378	-1.649632
32	H	4.800068	0.317990	-1.458628
33	H	3.851720	-0.957093	-2.238400
34	H	3.281161	0.718087	-2.268031
35	H	-3.046952	2.015328	-1.045352
36	H	-1.097099	3.285250	-0.048738
37	H	-1.690523	-0.077601	-1.579829
38	H	-0.967865	1.826801	2.285799
39	H	0.652666	0.964520	2.422125
40	N	-5.191904	-0.918242	-1.199590
41	H	-5.413965	-0.506248	-2.104150
42	H	-5.895236	-0.579393	-0.545844
43	H	-5.339131	-1.921346	-1.295014
44	H	-3.259215	-0.362210	-0.772273

****

TS-DE·NH_3 (transition state structure; imaginary frequency = –83.4)

1	C	0.067111	1.459322	0.507962
2	C	1.327257	1.047343	-0.198263
3	C	1.544081	-0.468105	-0.622569
4	C	-0.210541	1.082324	1.782442
5	C	0.854281	-1.533984	0.220810
6	C	-1.637616	-0.718893	0.366815
7	C	-0.665134	-1.769895	-0.065709
8	H	0.985159	-1.359657	1.292683
9	H	-0.932354	-2.704771	0.440237
10	H	-0.776760	-1.918955	-1.146286
11	C	3.082052	-0.264414	-0.304164
12	C	2.692914	0.990143	0.535082
13	H	1.331945	-2.495210	0.006076
14	H	1.333254	-0.663252	-1.681727
15	H	2.588615	0.746756	1.597224
16	H	3.327702	1.876885	0.445577
17	C	-0.941029	2.295638	-0.253539
18	C	-2.225968	0.181986	-0.643399
19	C	-2.349532	1.667411	-0.256487
20	H	-2.835489	1.773339	0.718291
21	H	-0.604077	2.430704	-1.285960
22	H	1.428854	1.660625	-1.096603
23	C	-2.391331	-0.975297	1.629120
24	H	-3.119699	-1.762908	1.370280
25	H	-1.761335	-1.380013	2.423832
26	H	-2.960176	-0.118231	1.988668
27	C	3.825726	-1.370179	0.447828
28	H	3.938599	-2.271884	-0.165277
29	H	4.833190	-1.025130	0.705595
30	H	3.332495	-1.652431	1.383315
31	C	3.854239	0.105245	-1.579126
32	H	4.864477	0.446655	-1.328682
33	H	3.949380	-0.763108	-2.240747
34	H	3.367040	0.905368	-2.146616
35	H	-2.987873	2.181744	-0.980464
36	H	-0.993543	3.298259	0.191242
37	H	-1.692175	0.092021	-1.594979
38	H	-1.093813	1.443131	2.298802
39	H	0.491701	0.515391	2.383211
40	N	-5.264051	-0.912302	-1.183658
41	H	-5.517527	-0.467703	-2.064244
42	H	-5.968308	-0.628286	-0.505237
43	H	-5.383442	-1.913799	-1.324170
44	H	-3.256543	-0.231303	-0.799252

****

E·NH_3 (minimum; frequency = 32.6)

1	C	0.390059	-0.913294	-0.424758
2	C	-1.039380	-1.092221	-0.475285
3	C	-1.818394	0.372377	-0.578085
4	C	1.070373	-0.249409	0.667433
5	C	-1.093227	1.597595	-0.023112
6	C	1.498962	1.163823	0.009082
7	C	0.281470	1.860529	-0.674731
8	H	-0.994956	1.547508	1.065631
9	H	0.486768	2.935997	-0.678424
10	H	0.226953	1.573508	-1.732748
11	C	-2.957935	-0.279861	0.290406
12	C	-1.952623	-1.377609	0.744610
13	H	-1.752962	2.451679	-0.215495
14	H	-2.077501	0.549108	-1.627395
15	H	-1.472026	-1.130267	1.694625
16	H	-2.325592	-2.404667	0.793438
17	C	1.210894	-1.184946	-1.634497
18	C	2.617874	0.884162	-1.032316
19	C	2.616212	-0.568047	-1.575352
20	H	3.235611	-1.189006	-0.921139
21	H	0.636527	-0.891350	-2.525390
22	H	-1.332680	-1.672403	-1.352553
23	C	2.012028	2.033671	1.170249
24	H	2.419417	2.969512	0.772069
25	H	1.211287	2.290038	1.871611
26	H	2.805341	1.525931	1.726615
27	C	-3.575857	0.568130	1.403333
28	H	-4.147857	1.408396	0.994952
29	H	-4.269799	-0.045827	1.987934
30	H	-2.831116	0.967042	2.097722
31	C	-4.065120	-0.850067	-0.608951
32	H	-4.752179	-1.467796	-0.021223
33	H	-4.648018	-0.042639	-1.064856
34	H	-3.669990	-1.473979	-1.418216
35	H	3.074130	-0.609533	-2.567185
36	H	1.276741	-2.284949	-1.713620
37	H	2.517210	1.599762	-1.855354
38	H	2.009066	-0.756233	0.954126
39	H	0.451586	-0.101385	1.551486
40	N	3.830235	-1.809388	1.859801
41	H	3.937797	-2.795199	1.627466
42	H	3.667236	-1.771291	2.864524
43	H	4.739790	-1.378743	1.703203
44	H	3.595559	1.073506	-0.578561

References

1. Zu, L.; Xu, M.; Lodewyk, M. W.; Cane, D. E.; Peters, R. J.; Tantillo, D. J. *J. Am. Chem. Soc.* **2012,** *134,* 11369–11371. doi:10.1021/ja3043245
